# Exploring how social media usage shapes self-presentation strategies among Saudi young adults

**DOI:** 10.3389/fpsyg.2025.1562917

**Published:** 2025-05-12

**Authors:** Ahmed Muyidi

**Affiliations:** Department of Media, King Saud University, Riyadh, Saudi Arabia

**Keywords:** self-presentation strategies, Saudi young adult, social media, psychological well-being, social media use

## Abstract

The rise of social networking sites (SNS) has changed how young people present themselves online to present idealized versions of their identities. These platforms enable selective self-presentation, shaped by features like anonymity and audience feedback. Especially in collectivist societies like Saudi Arabia, socio-cultural norms remain an effective factor, affecting online behaviors and leading to anonymous or semi-anonymous interactions. Despite the growing reliance on social networks, limited research addressed how these factors impact self-presentation strategies. This study examines how self-presentation on social media is affected by usage patterns among young adults in the Kingdom of Saudi Arabia. Using structured surveys, quantitative data are gathered from a sample of 715 young adults living countrywide. Results revealed that exemplification, ingratiation, and self-promotion are the most commonly used self-presentation strategies among Saudi youth. Negative usage patterns and a high number of followers used these strategies more frequently. Users disclosing users’ identities supported exemplification, reflecting cultural influences on online behaviors. Besides, increased time spent on social media was linked with greater use of all self-presentation strategies, highlighting potential risks to psychological well-being, i.e., anxiety and low self-esteem. Findings suggested a strong association between digital behaviors, audience dynamics, and impression management in a collectivist cultural context. Finally, cultural and clinical implications are presented, and limitations are highlighted.

## 1 Introduction

The rapid development of communication technologies and social network sites (SNSs) has had a significant influence on young adults and their social lives in cyberspace, providing them with more opportunities to present themselves to their community and the world (Ali et al., 2025; Li et al., 2021). These networks provide a variety of privileges to people who want to build desired characteristics and present selected information about themselves to manage feedback and other impressions (Ostic et al., 2021). One of the most notable studies to understand the concept of self-presentation was by Goffman (Miller, 1995). Self-presentation refers to “how people attempt to present themselves to control or shape how others (called the audience) view them” (Erin, 2021). Self-presentation is part of a broader set of behaviors called impression management. Impression management refers to “the controlled presentation of information about all sorts of things, including information about other people or events” (Boz and Guan, 2017). Thus, Goffman’s propositions are critical to this study as they address self-presentation and impression management in real social networks (Pasha, 2021). Tuten (2023) argued that social networking sites allow users to create and maintain profiles, other member identification and participate by consuming or producing content provided with their connections” (Tuten, 2023). Many young people are attracted to and show maximum presence on social media sites such as TikTok, Facebook, Instagram, YouTube, and Twitter (Patel and Binjola, 2020; Youssef et al., 2024), In other words, Social Networking Sites (SNS) enable people to present themselves in the way in which they prefer or assume as befitting their audiences (Aksar et al., 2023; Ali and Pasha, 2024).

Notably, these Social Networking Sites (SNS) offer self-presentation features easily adjustable to users, allowing them to present themselves selectively (Javornik et al., 2022). Furthermore, SNSs allow platform users to ‘switch between the authentic and (in)authentic self with character features of social media (Fioravanti et al., 2021). Moreover, they have allowed people to conceal undesirable attributes and highlight desirable ones higher than face-to-face communication by carefully optimizing the content before posting (Steinsbekk et al., 2021). In addition, Social Networking Sites (SNS) provide users with more opportunities for self-presentation and to get feedback on their self-presentation (Hanaysha, 2022). Accordingly, O’Day and Heimberg (2021) argued that since online communication differs from off-line, critics have continuously debated whether people reveal their true selves on social media, or whether they tend to present an idealized version of themselves. Online communication features like anonymity and reduced information richness have been argued to increase self-disclosure. Furthermore, asynchronicity, multiple audiences, and audience feedback have been claimed to favor self-presentation. Particularly in collectivist societies like Saudi Arabian culture, socio-cultural norms often shape how individuals present themselves online, leading to the widespread use of anonymous or partially anonymous accounts (Alshwiah and Alaulamie, 2022). Despite the growing reliance on social media, limited research focused on how these factors influence self-presentation strategies and their psychological implications (AlMuammar et al., 2021; Mohammed and Ferraris, 2021), further adding more relevance and necessity to the current study. Social media users are rated among the most active SNS users in Saudi Arabia. Especially, during and after the Covid19 pandemic, this usage has increased more (StatCounter, 2022). Saudi Arabia’s internet population exceeds 40 million people, of which 67.02% are considered young adults (under 35 years old) (Statista, 2022), thereby emphasizing the importance of studying the behavior of Saudi young adults on social media.

This paper mainly examines how specific characteristics, including patterns of use, number of followers, and account naming, relate to self-presentation strategies employed by young adults in Saudi Arabia. This study aims to address a significant gap in the existing literature during the current era of digitalization. Since the number of social media users has surged in the post-pandemic period, there is a notable difference between the current online population and that of the pre-pandemic era. Hence, this research fills the relevant technological gap, providing insights into how self-presentation has changed during the current era as compared to the pre-pandemic era. The significance of this study can be determined as it provides robust insights regarding social media use among the young generation. It also informs parents about the social media use among their children, patterns, and its potential impacts on their psychological well-being in cyberspace.

## 2 Research methods

### 2.1 Study design

This study involves a quantitative design, as the numerical data is collected quickly, with generalizable results (Apuke, 2017). A structured questionnaire is employed for data gathering adopted from existing literature. However, a minor formatting and editing of the questionnaire is performed to match the current study aims and problem. The data were gathered from June 2022 to October 2022. Once the data were gathered, all the responses were carefully evaluated and calculated. Later, data from the responses were analyzed using the statistical analysis software SPSS, including both descriptive and inferential statistics suggested by Habes et al. (2021).

### 2.2 Sampling approaches

The researcher used an online survey questionnaire to collect data from a sample of 715 Saudi undergraduate students (*N* = 715). The number of followers for this study was divided into six categories: no followers, fewer than 100, 100–500, 600–1,000, 2,000–10,000, and more than 10,000. The sample size of 715 was selected to ensure sufficient statistical power of subgroup analysis and generalizability. Although no specific population frame was available, the size exceeds the standard minimum for survey research with a 95% confidence level and a 5% margin of error, enhancing the reliability and validity of the results (Adam, 2020). The snowball sampling method was used, where initially recruited participants were used to recruit other participants. According to Alvi (2016), snowball sampling is ideal for accessing dispersed populations in survey-based studies. This approach also helps to ensure maximum participation for the population members, facilitating access to a broad and diverse sample across multiple institutions. The relevant method was used to reach Saudi undergraduates in Saudi colleges and universities who are active social media users through two approaches: First, professors and lecturers from different cities were contacted and asked to announce the study among their students. Second, the students who consented to participate were asked to forward the details to their colleagues. This combination of instructor recruitment and snowball sampling was used to increase the study’s sample size and reach active social media users.

### 2.3 Measures

#### 2.3.1 Self-presentation scale

A modified Revised Self-Presentation Scale (RSPS), which involves five strategies (ingratiation, self-promotion, intimidation, exemplification, and supplication), was used for the self-presentation strategies. The participants completed a self-presentation scale, which was revised by Lee et al. (1999) and then modified by Guan et al. (2025) to capture Saudi adolescents’ social media strategy usage. However, some sentences were slightly modified or rephrased to be more culturally appropriate and acceptable. This scale measures the five common strategies through some online practices such as:

1. Ingratiation: (i.e., On social media, I compliment people to get them on my side).
2. Self-promotion: (i.e., On social media, I point out the positive things I do that other people fail to notice).
3. Intimidation: (i.e., I threaten others on social media when I think it will help me get what I want from them).
4. Exemplification: (i.e., On social media, I try to set an example for others to follow).
5. Supplication: (i.e., I ask others to help me on social media sometimes).

A 5-point Likert scale was applied to the answers (from strongly agree. to strongly disagree).

#### 2.3.2 Usage pattern scale

Trifiro and Gerson (2019) found that in terms of Facebook, the models of passive and active use best fit the data when categorized into three subscales:

1. Active social use includes direct written communication between the user and their friends, such as posting and commenting on others’ posts.
2. Active non-social use refers to direct communication with no written content, such as liking, re-posting, liking, and RSVPing (replying or confirming attendance or acceptance of an invitation) to events.
3. Passive use: when users merely consume content without replying or interacting with others on social media.

Table 1 summarzies the social media usage patterns scale. Based on this categorization (Trifiro and Gerson, 2019), a brief scale of five statements to measure the patterns of social media use is applied in this study:

**Table 1:** Social media usage patterns scale.

Social media usage patterns	Questionnaire items	Strongly disagree 1	Disagree 2	Neutral 3	Agree 4	Strongly agree 5
Active social use (Direct written communication)	Usually, I write/publish from my account on social media					
	Usually, I write comments on other posts on social media.					
Active non-social (Direct unwritten communication)	Usually, I “like” other posts on social media					
	I do share or re-post (e.g., retweet) some content on social media					
Passive use (Consumption only)	On social media, I only explore and view content					

## 3 Results

The sample consisted of *N* = 715 Saudi undergraduates from all five main regions of Saudi Arabia and more than 40 public and private universities. The survey was sent to participants through the personal relations of students and staff members. The process of data collection took about 2 weeks in November 2022. The participants reported their gender, age, year of study, GPA, region of residence, and subjective socioeconomic status. Table 2 represents the summary of respondents’ demographics.

**Table 2:** Demographics of study respondents.

Variables	Constructs	Frequency	Percent
Gender	Male	214	29.9
	Female	501	70.1
	Total	715	100
Year of study	1st year	83	11.6
	2nd year	123	17.2
	3rd year	147	20.6
	4th year	175	24.5
	5th year	187	26.2
	Total	715	100
Discipline	Humanities and social	365	51
	Management	87	12.2
	Science	169	23.6
	Health and medicine	94	13.1
	Total	715	100
GPA/Grade	Excellent	348	48.7
	Very good	253	35.4
	Good	102	14.3
	Satisfactory	12	1.7
	Total	715	100
Region	Central	393	55
	Eastern	70	9.8
	Western	69	9.7
	Southern	131	18.3
	Northern	52	7.3
	Total	715	100
Socio-economic status	Low	83	11.6
	Middle	411	57.5
	High	221	30.9
	Total	715	100

Regarding their social media usage, the majority of the sample (51%) reported having fewer than 100 followers on their primary account, and only a small minority (1.8%) reported having more than 10,000 followers. A majority of the sample (85%) reported that their social media accounts were anonymous, either through the use of a fake name (17.5%) or only initials or part of their names (67.3%), and only 15% reported that their accounts displayed their real names. Increased use of anonymous account names was linked to strict cultural factors within Arab societies that practice collectivism and follow group rules that require obedience and loyalty. Regarding the duration of time spent on social media, only 15.2% stated that they use social media for less than 1 hour a day, half of the sample (50.9%) reported using it between 2 and 5 hours daily, and 40.3% reported using it more than 5 hours daily. Table 3 represents the summary of responses regarding social media usage.

**Table 3:** Social media usage.

Variables	Valid	Frequency	Percent
Number of followers	Fewer than 100 followers	369	51.6
	100–500 followers	232	32.4
	600–1,000 followers	72	10.1
	2,000–10 k followers	29	4.1
	More than 10 k	13	1.8
	Total	715	100
Account name	Use of real name	109	15.2
	Part of name	481	67.3
	Fake name	125	17.5
	Total	715	100
Time (duration) spent on social media	Low	63	8.8
	Medium	364	50.9
	High	288	40.3
	Total	715	100

The results also show that almost half of the sample (49.7%) reported being negative in their pattern of usage, while 22.4% were positive but non-social. Furthermore, only 28% were positive and social on social media. Table 4 shows the summary of results regarding patterns of social media usage.

**Table 4:** Patterns of social media usage.

Variables	Valid	Frequency	Percent
Pattern of usage	Positive social	200	28
	Positive non-social	160	22.4
	Negative	355	49.7
	Total	715	100

Table 5 summarizes the results regarding Self-Presentation Strategies Used by the Respondents. The most common strategy was exemplification (mean = 3.31), followed by ingratiation and self-promotion. The least reported strategies were supplication and intimidation.

**Table 5:** Self-presentation strategies used by the respondents.

Type of self-presentation strategy	Mean	Std. deviation	Response rate	Rank
Exemplification	3.31	1.044	Medium	1
Ingratiation	2.74	1.121	Medium	2
Self-promotion	2.55	1.112	Medium	3
Supplication	2.33	1.123	Low	4
Intimidation	1.85	1.206	Low	5

*RQ1*: What is the degree of relation between the self-presentation strategies used by the sample and their pattern of usage on social media?

*H1*: There is a significant correlation between the self-presentation strategies used by the sample and their pattern of usage on social media.

To examine the differences in the responses about the self-presentation strategies of the sample and their usage pattern, a one-way analysis of variance (ANOVA) is recommended for quantitative studies (Howes and Chapman, 2024) was conducted. The table presents the results of ANOVA concerning the relation between self-presentation strategies and usage patterns. According to the *F* value and the significance levels presented in Table 6, there are statistically significant differences at the 0.05 level between the average participant responses concerning their social media self-presentation strategies and usage patterns. Regarding the partial *η*^2^, results indicate that self-presentation strategies have the highest effect size with partial *η*^2^ 0.741, ingratiation has the second highest effect size *η*^2^ = 0.540, and supplication remains the third highest with the partial *η*^2^ value of 0.381.

**Table 6:** ANOVA test of the relation between self-presentation strategies and usage pattern.

Dependent variables		Sum of squares	DF	Mean square	*F*	Partial *η*^2^	Sig.
Exemplification	Between groups	47.299	2	23.650	23.041	0.356	0.000
	Within groups	730.806	712	1.026			
	Total	778.105	714				
Self-promotion	Between groups	124.662	2	62.331	58.539	0.741	0.000
	Within groups	758.123	712	1.065			
	Total	882.785	714				
Ingratiation	Between groups	104.954	2	52.477	47.177	0.540	0.000
	Within groups	791.990	712	1.112			
	Total	896.944	714				
Intimidation	Between groups	88.516	2	44.258	33.155	0.367	0.000
	Within groups	950.429	712	1.335			
	Total	1038.944	714				
Supplication	Between groups	87.933	2	43.966	38.547	0.381	0.000
	Within groups	812.090	712	1.141			
	Total	900.023	714				

To further confirm these findings, an LSD test was conducted. The analysis indicated that participants who followed a negative usage pattern used the exemplification strategy more frequently than those with positive social or non-social patterns. Besides, self-promotion and ingratiation strategies were also significantly more prevalent among participants, showing a negative pattern of social media use. The intimation strategy was found to be more commonly employed by those with a negative pattern. Finally, the supplication strategy, which involves self-deprecation to elicit support or sympathy, was also significantly higher among the negative pattern users than those in the positive usage categories.

*RQ2*: To what extent do self-presentation strategies used by the sample differ or agree with their follower numbers on social media?

*H2*: There is a significant correlation between self-presentation strategies used by the sample and follower numbers on social media.

An ANOVA was conducted to determine the differences in the participants’ responses regarding their self-presentation strategies on social media and the number of followers they had. Table 7 presents the results of the ANOVA test assessing differences in participants’ social media self-presentation strategies about their number of followers. Based on the *F*-values and significance levels. The statistically significant differences were found at the 0.05 level. To examine specific group differences, the LSD test was conducted. The calculation of partial *η*^2^ indicates that exemplification and self-promotion showed statistically significant differences with large effect sizes (*η*^2^ = 0.311 and *η*^2^ = 0.725, respectively). Ingratiation also showed a significant result with a moderate effect size (*η*^2^ = 0.240). While intimidation and supplications have small effect sizes (*η*^2^ = 0.44 and *η*^2^ = 0.052).

**Table 7:** ANOVA test of the relationship between self-presentation strategies and number of followers.

Dependent variables		Sum of squares	DF	Mean square	*F*	Partial *η*^2^	Sig.
Exemplification	Between groups	60.205	4	15.051	14.886	0.311	0.000
	Within groups	717.901	710	1.011			
	Total	778.105	714				
Self-promotion	Between groups	96.349	4	24.087	21.746	0.725	0.000
	Within groups	786.435	710	1.108			
	Total	882.785	714				
Ingratiation	Between groups	65.222	4	16.305	13.919	0.240	0.000
	Within groups	831.722	710	1.171			
	Total	896.944	714				
Intimidation	Between groups	41.946	4	10.487	7.468	0.044	0.000
	Within groups	996.998	710	1.404			
	Total	1038.944	714				
Supplication	Between groups	39.053	4	9.763	8.051	0.052	0.000
	Within groups	860.970	710	1.213			
	Total	900.023	714				

Findings further revealed that the exemplification strategy was more commonly used by participants with more than 100 followers, especially with 600 to 10,000 followers, compared to those with fewer than 100 followers. Furthermore, social media users with 100 or more followers used self-promotion strategies more often than those with fewer than 100. The ingratiation strategy also followed this trend, being significantly more common among those with over 100 followers, particularly among participants in the 2000 to 10,000 category used it more than individuals with fewer than 1,000 followers. The ingratiation strategy further followed this trend. It was significantly more common among individuals with over 100 followers, particularly among participants in the 2000 to 10,000 category, who use it more than people with fewer than 1,000 followers. Regarding intimidation, the strategy was significantly more prevalent among respondents with 2000 to 10,000 followers as compared to those with fewer than 1,000 and also more frequent among those with 100 to 500 followers relative to those with fewer than 100. Finally, using the supplication strategy was found to be higher among respondents with 2000 to 10,000 followers and those with more than 10,000 followers, as compared to respondents in all other follower categories.

*RQ3*: What is the correlation between the self-presentation strategies used by the sample and their account name on social media?

*H3*: There is a significant correlation between the self-presentation strategies used by the sample and their social media account name.

An ANOVA was conducted to determine the differences in the participants’ responses about their social media self-presentation strategies and account names. Table 8 represents the ANOVA test analyzing differences in participants’ self-presentation strategies based on the type of account name used on social media platforms. The relevant analysis indicated no statistically significant differences at the level 0.054 in the strategies of self-promotion in graduation, intimidation, and supplication regarding account name categories. Regarding the effect size, only the “exemplification” strategy indicates a statistically significant result with a small effect size of *η*^2^ = 0.010. All other strategies did not reach statistical significance, having a smaller effect size.

**Table 8:** ANOVA test of the relation between the self-presentation strategies and account name.

Dependent variables		Sum of squares	DF	Mean square	*F*	Partial *η*^2^	Sig.
Exemplification	Between groups	7.846	2	3.923	3.626	0.010	0.027
	Within groups	770.259	712	1.082			
	Total	778.105	714				
Self-promotion	Between groups	3.282	2	1.641	1.328	0.004	0.266
	Within groups	879.503	712	1.235			
	Total	882.785	714				
Ingratiation	Between groups	2.554	2	1.277	1.017	0.003	0.362
	Within groups	894.390	712	1.256			
	Total	896.944	714				
Intimidation	Between groups	5.633	2	2.816	1.941	0.005	0.144
	Within groups	1,033.312	712	1.451			
	Total	1,038.944	714				
Supplication	Between groups	1.331	2	0.666	0.527	0.001	0.590
	Within groups	898.692	712	1.262			
	Total	900.023	714				

To further affirm these differences, an LSD test was conducted. The findings showed that participants who used either part of their real name or full name used the exemplification strategy more often than those who used anonymous account names. This implies that individuals who disclose their identity are more likely to engage in online behavior aimed at showing integrity, morality, or accountability through their social media presence.

*RQ4*: To what degree do the self-presentation strategies used by the sample relate to the duration of time spent on social media?

*H4*: There is a significant correlation between the self-presentation strategies used by the sample and the duration of time spent on social media.

An ANOVA was conducted to examine the differences in the social media self-presentation strategies and duration of use. Table 9 presents the results of the ANOVA test examining the relationship between participants’ self-presentation strategies and their duration of social media usage. The *F* values and associated significance levels indicate statistically significant differences at 0.05 across all self-presentation strategies about usage duration. To investigate these differences further, an LSD test was conducted. The ANOVA’s effect size (partial *η*^2^) indicates small to moderate effects across all self-presentation strategies. The highest effect is observed for self-promotion (*η*^2^ = 0.025), followed by ingratiation (*η*^2^ = 0.024) and supplication remains whoring the third highest effect size (*η*^2^ = 0.017). The results revealed that the exemplification strategy was significantly more preventive among participants reporting medium to high usage duration than those with low usage. A similar pattern emerged for the self-promotion strategy, where individuals with medium to high social media use engaged in self-promotional behaviors more frequently than those with limited usage. The ingratiation strategy also showed higher use among respondents with medium to high durations of social media activity, suggesting a greater tendency to seek social approval among more frequent users. For the intimation strategy, differences were most pronounced in favor of those reporting high usage, who used this strategy more frequently than those in the low and medium categories. Besides, the supplication strategy was significantly higher among high-duration users than among medium and low-duration users. These results indicate a consistent trend: increased social media engagement is positively linked with the frequent use of self-presentation strategies across the board.

**Table 9:** ANOVA test of the relation between the self-presentation strategies and duration of use.

Dependent variables		Sum of squares	DF	Mean square	*F* ^2^	Partial *η*^2^	Sig.
Exemplification	Between groups	11.527	2	5.764	5.353	0.015	0.005
	Within groups	766.578	712	1.077			
	Total	778.105	714				
Self-promotion	Between groups	22.342	2	11.171	9.244	0.025	0.000
	Within groups	860.442	712	1.208			
	Total	882.785	714				
Ingratiation	Between groups	21.808	2	10.904	8.871	0.024	0.000
	Within groups	875.135	712	1.229			
	Total	896.944	714				
Intimidation	Between groups	9.245	2	4.623	3.196	0.009	0.041
	Within groups	1,029.699	712	1.446			
	Total	1,038.944	714				
Supplication	Between groups	15.060	2	7.530	6.058	0.017	0.002
	Within groups	884.963	712	1.243			
	Total	900.023	714				

## 4 Discussion

This study highlighted a novel perspective concerning digital psychology by highlighting how cultural norms, anonymity, follower count, and patterns of social media use intersect to shape self-presentation strategies among Saudi youth. While previous research (See Gakahu, 2024; Luebke and Steffan, 2025; Trysnes and Synnes, 2022) has established general associations between social media behaviors and psychological outcomes, this research deepens the understanding by revealing that individuals who engage in “negative usage patterns indicate a broader and more intense use of self-presentation strategies. This study demonstrated that exemplification is supported when users disclose their real identity, suggesting a culturally rooted psychological mechanism for managing social perception and reputation in Saudi Arabia.

The findings specified that exemplification, ingratiation, and self-promotion were the participants’ most frequently used self-presentation strategies. This finding corroborates the results of Erin (2021), in which a review of different studies indicated that adolescents reported using the exemplification strategy, followed by ingratiation and self-promotion. Moreover, the findings show that there were statistically significant differences between the participants’ average social media self-presentation strategies and the usage patterns. The participants who used the negative pattern, who published, posted, or communicated less frequently than others on social media, employed more self-presentation strategies than others. Negative patterns here include any form of posts, content, statements, or anything that would suggest failure, weakness, powerlessness, abuse, controversy, and out of norms. They used exemplification, self-promotion, ingratiation, intimidation, and supplication more often than those engaging in positive usage patterns. Notably, Hjetland et al. (2024) witnessed an increased emphasis on self-presentation, characterized by behavior including feedback, strategic self-presentation, and increased social comparisons, which was linked to increased symptoms of depression and anxiety among both males and females. Chu et al. (2023) also found that online self-presentation leads to social relationships and support. However, excessive self-presentation leads to increased anxiety and reduced mental well-being. These findings recommend that mental health practitioners and digital well-being educators should develop targeted interventions to help young users recognize and manage excessive self-presentation behaviors to reduce risks of anxiety and social comparisons.

The current study results further raise the question of whether these participants’ negative pattern of social media use indicates a degree of low self-esteem, propelling them to try additional strategies to be accepted by others. This result could be compared to the findings of Cheng et al. (2019), that participants with an impersonal orientation utilized supplication strategies to present a weak helpless image in online relationships. This could be due to their need to acquire attention and sympathy from others, leading them to express themselves through changing online strategies (Bowman-Smith et al., 2021). Participants with negative feelings about themselves sometimes use their weakness and helplessness by employing supplication tactics on social networking sites (Valdés-Cuervo et al., 2021). Linking the current study results with the psychological realms, Mann and Blumberg (2022) also witnessed negative social media usage linked with lower self-esteem and lower confidence, leading to adopting false or inconsistent selves. The results of current study and the study by Mann and Blumberg (2022) highlighted and emphasized a positive social media usage to ensure the positive outcomes on overall mental health and psychological well-being. Existing studies also support the idea of positive social media usage among young adults to improve their self-esteem (Valkenburg and Beyens, 2021) and body image (Vall-Roqué et al., 2021). Based on the relevant findings, it is suggested that educators and clinicians should focus on building self-esteem programs that may help young users develop a stable self-concept and reduce reliance on validation through supplication strategies. Promoting positive and purposeful social media use can foster healthier psychological outcomes.

Another important result is that there were statistically significant differences between the strategies of self-presentation that the participants used on social media and their follower numbers. The participants with more followers used more self-presentation strategies than those with fewer than 100 followers. This suggests that self-presentation strategies were associated with the audience either watching or following an individual. Without an audience, individuals do not try to present themselves; however, with a large audience, they aim to continuously satisfy this audience while continuously presenting themselves in varying ways in a competitive environment such as that of SNSs. Moreover, different audience segments have different expectations about their public image (Miller, 1995). SNS users must adjust their public image to the expectations of all these groups, leading them to try out different self-presentation strategies according to the circumstances (Tuten, 2023). In their study, Sciara et al. (2023) also found that more young Italian social media users employ more self-presentation strategies with more followers. Using these social media platforms for self-presentation increases users’ drive to manage impressions, leading to more frequent and different tactics. This behavior is rooted in the psychological need for social approval and the desire to meet perceived audience expectations. These results recommend that platform’ designers and digital policy-makers consider introducing tools that help users manage audience segmentation and reduce performance pressure to promote healthy self-presentation practices.

The third finding is that there was no statistically significant difference between the self-presentation strategies (self-promotion, ingratiation, intimidation, and supplication) used by the participants on social media and their account names. Nevertheless, there were statistically significant differences between the exemplification strategy and the account name. The differences in the exemplification strategy indicate that the participants who used their actual names or part thereof on social media employed the strategy of exemplification more frequently than those who maintained anonymity. This result indicates that some strategies, such as intimidation and supplication (and sometimes self-promotion), are not favorable in the local culture of Saudi Arabia, and using them with a real name is not advantageous to a person’s reputation. Thus, exemplification is the safest and most reasonable strategy when one’s real name is used. A study by Alshehri (2023) also back the current findings, indicating that pseudonymity offers users the dual benefits of “no strings attached” and “no questions asked” to young Saudi Twitter users. It enables them to manage the complexities of self-presentation and self-disclosure in a collectivist society. While anonymity provides freedom, the audience’s presence still significantly influences users’ online behaviors, including self-presentation strategies. Digital platforms working in collectivist cultures should provide subtle identity display options to allow users to manage self-presentation effectively. They can acquire greater flexibility, balancing authenticity, privacy, and reputational concerns.

The last significant finding of this study is that there were statistically significant differences between the strategies of self-presentation used by the participants on social media and the duration of time spent on social media. The participants who used social media for a high degree of time used all the self-presentation strategies more than those whose average social media use was low to medium. This result emphasizes further investigation to examine the direction of this relation. This would deal with whether those who use social media to a greater extent are affected by a digital common culture of self-presentation, thereby mimicking others, or whether those who tend to care about their self-image are more eager to spend their time online to shape an idealized image of themselves (Hoffner and Bond, 2022). Skogen et al. (2021) also supports these results, as they found a higher focus on self-presentation linked with increased symptoms of anxiety and depression among Norwegian youth. Besides, the correlations between self-presentation and decreased life satisfaction were also found. Therefore, the intensity of self-presentation behaviors played a critical role in these psychological outcomes. Finally, it is recommended that mental health practitioners should develop awareness programs that highlight psychological consequences and prolonged social media use. These platforms can introduce usage nudges to promote mindful engagement, especially targeting the young generation.

## 5 Clinical and cultural implications

This study provides important clinical and cultural insights into how young adults in Saudi Arabia use social media for self-presentation, emphasizing its psychological and behavioral implications. The results revealed a predominant reliance on anonymous identities and a high frequency of social media use, often exceeding 5 hours daily. These patterns are linked with distinct self-presentation strategies, particularly exemplifications, self-promotion, and ingratiation, which are more commonly employed by users with negative usage patterns and larger follower accounts. Clinically, this suggests that excessive or anonymous social media engagement may reinforce special behavioral strategies associated with self-esteem, identity formation, and social comparison, factors typically linked with anxiety, low self-confidence, and other mental health outcomes. Furthermore, this study highlights the influence of cultural norms, where anonymity may serve as a coping mechanism against social expectations and pressures. The significant correlations between follower count, usage duration, and self-presentation strategies suggest that digital social validation could shape individuals’ self-concepts, possibly affecting their psychological well-being. Besides, the preference for anonymous accounts may reflect deeper concerns around privacy, fear of judgment, and self-censorship, which can hinder authentic self-expression and contribute to internal stress. The results of this study can help clinicians recognize how social media platforms may act as both a coping space and a source of psychological stimulation. Understanding these insights is critical for developing culturally appropriate therapeutic strategies that consider digital identity m, management. Integrating the acquired insights about online behavior into mental health evaluations may provide a more comprehensive view of clients’ social and emotional lives. Overall, this study contributes to the broader understanding of digital behavior in non-western, culturally conservative societies and can inform targeted mental health interventions, digital literature programs, and culturally sensitive counseling approaches that address identity expression and digital socialization among young people in the Arab world.

## 6 Conclusion

This study contributes to the understanding of online self-presentation and its relationship to the features of social media usage. The findings of this study provide evidence that self-presentation is related to several factors and that how individuals use social media affects how they present themselves. The results show that the pattern of use, number of followers, account name anonymity, and duration of use were all important factors relating to the self-presentation strategies used by individuals on SNSs and how they present themselves to the world. The study delivers significant findings about the importance of these factors, how they affect young adults’ image of themselves, and how they behave with others on SNSs. Although the study is limited by the sample’s size and geographical and cultural features, it presents many directions for future research. Each variable of social media usage should be further examined regarding self-presentation strategies and different sample segments around the world.

### 6.1 Limitations

Despite this study has filled an important gap in the existing literature, it has some limitations. First, it involves a single-method, quantitative design, which limits its scope. The second limitation involves a lack of theoretical underpinnings. The final imitation involves using a descriptive approach rather than a critical analysis. Future researchers can consider these limitations and conduct more studies on the relevant phenomena to delimit the scope of current research.

## Data Availability

The original contributions presented in the study are included in the article/supplementary material, further inquiries can be directed to the corresponding author.
